# Hybrid machine learning for SCC strength prediction using metaheuristic optimization

**DOI:** 10.1038/s41598-026-51974-1

**Published:** 2026-05-20

**Authors:** Akhilendra Sharma, Rahul Biswas, Sharad Singh, Manish Kumar, Nadia Razali

**Affiliations:** 1 Department of Applied Mechanics, Visvesvaraya National Institute of Technology, Nagpur, India; 2 Department of Civil Engineering, SRM Institute of Science and Technology (SRMIST), Deemed to be University, Tiruchirappalli, Tamil Nadu India; 3 Malaysian Institute of Chemical and Bioengineering Technology (UniKL MICET), Universiti Kuala Lumpur, Lot 1988, Kawasan Perindustrian Bandar Vendor, Taboh Naning, 78000 Alor Gajah, Melaka Malaysia

**Keywords:** Self-compacting concrete, Machine learning, Gradient boosting, Metaheuristic optimization, Compressive strength prediction, SHAP analysis, Engineering, Mathematics and computing

## Abstract

Self-Compacting Concrete (SCC) represents a significant innovation in modern concrete technology owing to its excellent flowability, workability, and mechanical performance. Accurate prediction of SCC compressive strength is essential for optimizing mix design, minimizing experimental effort, and promoting sustainable material usage. In this study, a hybrid Machine Learning (ML) framework was developed by integrating Gradient Boosting (GB), Adaptive Boosting (ADA), and Random Forest (RF) models, optimized through metaheuristic algorithms such as Grey Wolf Optimizer (GWO), Mountain Gazelle Optimizer (MGO), Brown Bear Optimizer Algorithm (BBOA), and Fox Optimizer (FO). A dataset containing 691 SCC samples with varied mix proportions and curing conditions was utilized for model development and validation. Statistical analysis using the coefficient of determination (R^2^), Root Mean Square Error (RMSE), Weighted Mean Absolute Percentage Error (WMAPE), and Willmott’s Index of Agreement (WI) revealed that the BBOA-GB model exhibited the highest predictive accuracy, with R^2^ values of 0.9955 and 0.9645 for training and testing, respectively. SHapley Additive exPlanations (SHAP) analysis indicated that slag content, cement content and curing age were the most influential factors affecting compressive strength. The proposed hybrid approach demonstrated improved performance compared to the considered baseline ensemble models. The novelty of this research lies in the comparative benchmarking of multiple metaheuristic–ensemble integrations and the development of a Graphical User Interface (GUI) for practical SCC strength prediction. The study contributes a reliable and efficient data-driven framework that enhances decision-making in SCC design and supports the advancement of sustainable concrete technology. However, the study is limited by the use of literature-based datasets and a restricted range of mix compositions. Future research will focus on incorporating larger and real-time datasets, exploring deep learning approaches, and extending the framework toward sustainability-driven mix optimization.

## Introduction

Concrete is the most widely used material in modern construction due to its strength and versatility. However, placing and compacting concrete in complex formworks, for mass concreting and underwater structures, is difficult and labor-intensive. The construction industry constantly seeks innovative materials and techniques to improve efficiency, durability, and sustainability. Self-Compacting Concrete (SCC) is a prime example, offering superior flowability and compaction without the need for mechanical vibration^1,2^. This unique property makes SCC highly desirable for complex structures and heavily reinforced sections where traditional concrete would struggle to fill all voids adequately^3^. SCC can produce a more homogeneous and denser microstructure resisting ingress whereas high workability of SCC reduces the risk of honeycombing and voids, thereby enhancing the performance of SCC, particularly its compressive strength, flexural strength, and durability^4^. Overall quality of the concrete is enhanced with reduced risk of segregation and honeycombing^5^. The ability of the mix to flow freely under its own weight imparts the Self-Compacting nature to SCC. Use of supplementary cementitious materials (SCMs) like fly ash, silica fume, and ground granulated blast furnace slag (GGBFS) enhance durability and reduce heat of hydration^6^. Predicting the compressive strength of concrete is a critical aspect of structural design and quality control. While traditional methods to determine compressive strength involving destructive testing, remain labour-intensive, time-consuming and costly, there is a growing need for non-destructive techniques that can provide rapid, reliable estimates of concrete properties in situ^7,8^. Numerous experimental works have been conducted to examine the impact of various factors, such as the proportion of cement replaced by SCMs, the age of the specimen during testing, and the ratio of aggregates to binder, on the compressive strength (CS) of concrete along with analytical studies to simulate the strength behaviour of concrete^9^. Furthermore, statistical models have been developed to predict the CS. However, the vast number of variables and complexity of the subject make it difficult to incorporate all parameters into these formulas, rendering them specific to certain materials and not generally applicable. Current tools for predicting the CS of SCC reflect this challenge, where empirical correlations are suggested to account for the effects of a small number of parameters. There is a need for a more resourceful and cost-effective method to estimate the CS of concrete and cement-based materials^10^.

Application of soft computing and machine learning (ML) techniques to real-world problems has grown substantially because of their exceptional capacity to detect non-linear and unclear correlations between a dataset’s input and output variables^11^. ML techniques such as artificial neural networks (ANN), decision trees (DT), support vector machines (SVM), genetic engineering programming (GEP), and deep learning (DL) are used to estimate various civil engineering problems^12,13^. One such application is to improve the SCC properties using ML based predictive models. The use of machine learning for predicting the CS of SCC presents transformative potential for the construction industry^14^. Techniques such as neural networks, support vector machines, and fuzzy logic have demonstrated significant potential for handling complex datasets and providing accurate predictions^15^. Artificial Neural Networks (ANNs) effectively model nonlinear relationships between mix proportions and strength, yielding R^2^ values exceeding 0.9 in most studies^16^. Kernel-based approaches like Support Vector Machines (SVM) address high-dimensional data, providing robust predictions for diverse mix designs^17^, whereas Ensemble Model techniques such as Random Forests and Gradient Boosting improve accuracy and handle noise in experimental datasets^18^. Compressive strength of ternary blended SCC with MP and rice husk ash has been predicted using multiple ML models^19^. Efficient SCC mixture compositions and compressive strengths were part of the extensive dataset used to train the ANN model^20^. Moradi et al. studied predicting the CS of metakaolin (MK)-contained concrete with different properties using ANN^21^. Asteris et al. used ANN to reveal the nature of MK based concrete^22^. To predict the CS of concrete, Özcan et al. presented comparative results of prediction outcomes of neural network and fuzzy logic paradigms for the long-term CS of silica fume-based concrete^23^. Sarıdemir used ANNs and fuzzy logic for predicting CS of concretes and mortars containing MK and silica fume^24^. Gilan et al. suggested an SVR-PSO paradigm for the prediction of CS of MK-contained concretes^25^. Huang et al. predicted the CS of cement-based materials with MK used on the hybrid ML paradigm^26^. Although the aforementioned ML paradigms achieved satisfactory results in predicting the CS of concrete and cement, they have multiple limitations such as uncertainty, time consumption, and local minima trapping issues. Thus, it is necessary to develop a high-performance ML paradigm, especially for estimating the CS of SCC^2^. Civil engineering optimization commonly uses meta-heuristic algorithms to optimize data mining models for ML algorithm hyperparameters. Thus, training ML models requires choosing the right meta-heuristic algorithm. Finding effective solutions and improving the precision of regression algorithms have been widely achieved in recent years through the use of optimization algorithms based on neural networks^27^.

The machine learning models selected in this study, namely RF, ADA and GB, were chosen due to their strong capability in capturing nonlinear relationships and their effectiveness for tabular engineering datasets. These ensemble models provide improved generalization, reduced overfitting and higher stability compared to single models such as artificial neural networks and support vector machines. In addition, their compatibility with metaheuristic optimization techniques enables efficient hyperparameter tuning, thereby enhancing predictive performance. Unlike previous studies that focus on single optimizer–model combinations, this study provides a systematic benchmarking of multiple metaheuristic–ensemble integrations under a consistent dataset and evaluation framework, ensuring fair comparison and reliable performance assessment.

## Research objective

The application of Gradient Boosting (GB) for predicting the compressive strength of self-compacting concrete (SCC) has been relatively limited in existing studies. As a regression tree-based ensemble method, GB is well-suited for capturing complex nonlinear relationships among input variables; however, its performance is highly dependent on the selection of optimal hyperparameters. Appropriate tuning of these parameters significantly enhances model robustness and predictive accuracy. In this study, a hybrid modeling framework is developed by integrating GB, AdaBoost (ADA), and Random Forest (RF) models with metaheuristic optimization algorithms, including Grey Wolf Optimizer (GWO), Mountain Gazelle Optimizer (MGO), Brown Bear Optimization Algorithm (BBOA), and Fox Optimizer (FO). This integrated approach enables efficient exploration of the hyperparameter space and leads to improved predictive performance compared to conventional standalone models. The novelty of this work lies in the systematic comparative evaluation of multiple metaheuristic–ensemble hybrid configurations rather than the development of a new algorithm. Furthermore, SHapley Additive exPlanations (SHAP) analysis is employed to interpret model predictions and identify the most influential input parameters affecting SCC strength. To enhance practical applicability, an open-source graphical user interface (GUI) is also developed, providing an accessible and user-friendly platform for real-time strength prediction and mix design optimization..

## Selected ML models

**Fig. 1:**
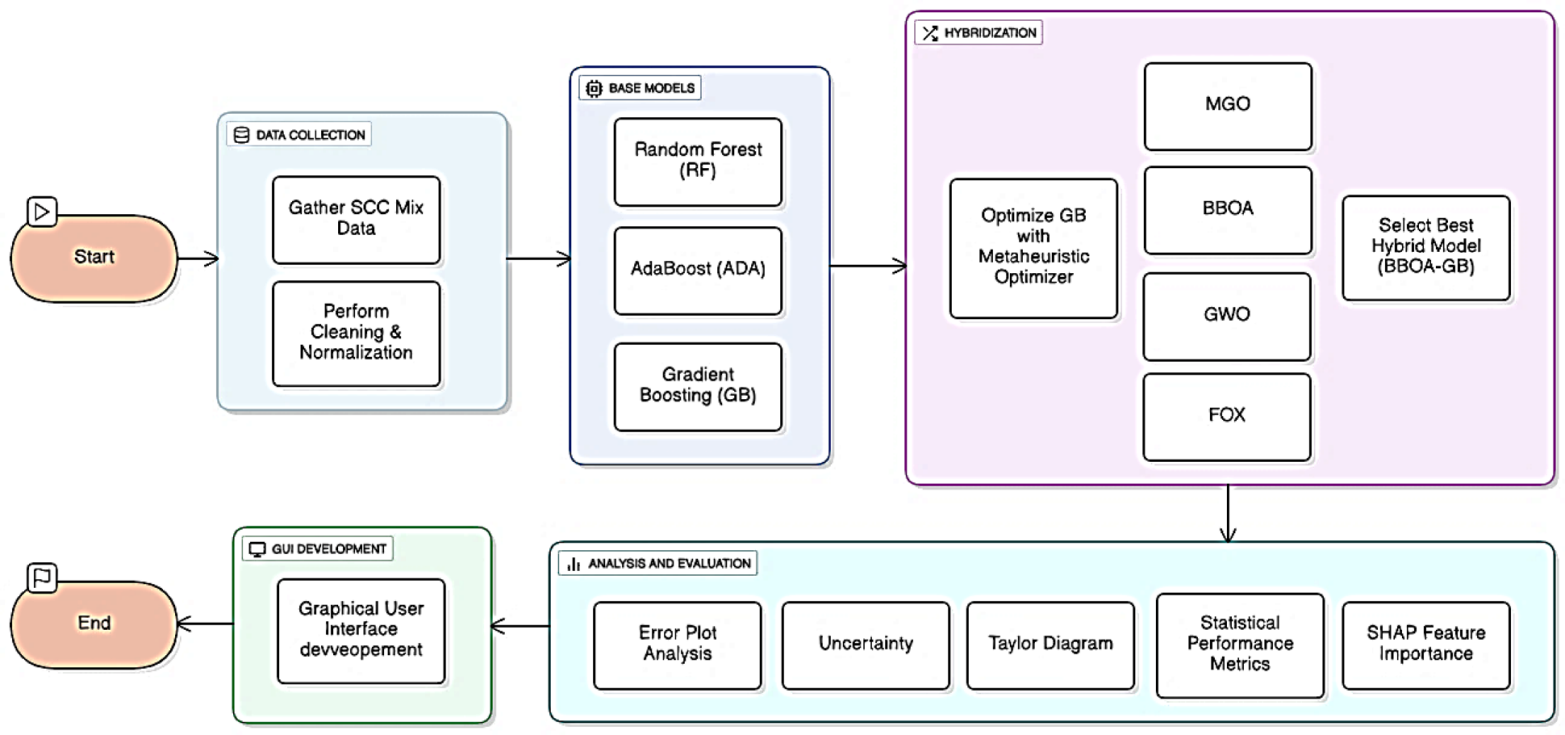
Workflow of the proposed hybrid ML framework for SCC strength prediction.

Figure 1 presents the overall workflow adopted for the development of the hybrid machine-learning framework for predicting the compressive strength of SCC. The procedure was initiated with data collection and preprocessing, during which SCC mix data obtained from published sources were cleaned and normalized to ensure consistency and uniform representation. In the subsequent stage, ensemble base models comprising RF, ADA and GB were developed to establish the baseline predictive performance. The GB model was then optimized using four metaheuristic algorithms—MGO, BBOA, GWO and FOX—to generate hybrid variants and identify the best-performing configuration (BBOA-GB). The optimized models were evaluated using multiple statistical performance indices, error-plot analysis, Taylor-diagram comparison, uncertainty assessment and SHAP-based feature-importance interpretation. Finally, a GUI was designed to facilitate user interaction and real-time prediction of SCC compressive strength.

### Random forest (RF)

Random Forest (RF) is an ensemble learning algorithm renowned for its remarkable accuracy in both classification and regression tasks. It consists of numerous decision trees that are constructed using a technique known as bootstrap aggregating (bagging). This method helps to mitigate overfitting, because it trains each tree on a randomly selected subset of the data. Developed by Breiman, the RF model employs two levels of randomness: first, it resamples the original dataset with replacement to generate multiple training datasets and second, it randomly chooses a subset of features at each node split. Each decision tree in the forest operates independently and the final prediction in regression tasks is obtained by averaging the outputs. The Classification and Regression Tree (CART) method is frequently utilized within RF to evaluate impurity and determine the optimal splits. However, the algorithm also utilizes out-of-bag (OOB) data for validation, which reduces the necessity for separate test datasets while still ensuring efficiency. Moreover, RF is remarkably scalable (which makes it apt for extensive datasets) without incurring significant computational expenses. By aggregating predictions that stem from multiple trees, Random Forest (RF) improves generalization and assures robust performance across various machine learning applications. However, this method proves effective because it integrates diverse viewpoints. Although it might appear intricate, the fundamental principle is quite simple: combining the outputs yields superior overall results.

### Ada boost (Ada)

AdaBoost is a powerful ensemble learning method that greatly improves predictive accuracy by combining several weak learners into a unified model. It operates through an iterative mechanism (notably) in which weak decision trees are trained; this process entails modifying the weight distribution of training examples based on their prediction errors. Misclassified examples are given increased weights in later iterations; this ensures that the model concentrates on more difficult instances. However, some may argue that this approach could lead to overfitting, but the effectiveness of AdaBoost often outweighs such concerns. The ultimate prediction is obtained from a weighted average of all weak learners, which improves generalization. Its ability to adaptively relay gradient information makes it resilient against outliers and irrelevant data, thus enhancing reliability in regression applications. However, AdaBoost may still encounter limitations when dealing with complex datasets, because of its general effectiveness.

### Gradient boosting (GB)

The gradient boosting regressor is a strong ensemble learning method that builds predictive models by iteratively minimizing a differentiable loss function. Originally suggested by Friedman, the GB model constructs trees in a stage-wise fashion, with each new tree being fitted to the residuals of prior iterations to correct mistakes made by earlier models. This method uses decision trees as weak learners to improve prediction accuracy by focusing on the negative gradients of the loss function. Gradient Boosting (GB) offers several benefits, particularly its capacity to reduce overfitting and make efficient use of computational resources. This is primarily accomplished by adjusting parameters such as the number of trees and the learning rate. The algorithm’s structure also improves interpretability by emphasizing the importance of various features, making it suitable for both regression and classification tasks. GB’s methodical approach to boosting predictive performance has established it as a prominent technique in the field of machine learning.

## Metaheuristic optimisation techniques for hybrid GB

### Grey wolf optimizer

Grey *Wolf Optimizer (GWO) was first introduced by Mirjalili *et al*.*^28^* and it mimics grey wolves display leadership and cooperate to hunt in nature. It separates the* candidate solutions into alpha (best), beta (second-best), delta (third-best) and omega (resting within the remaining solutions). During the search process, wolves are made to behave like they would in the wild by encircling targets, hunting and attacking and their locations are updated according to suggestions from the top three leaders (alpha, beta, delta). This way of organizing allows the algorithm to look for and use the best solutions. GWO *is chosen* due to its simple structure, small number of parameters and its skills in getting away from local maxima. This study used GWO to adjust the values of important parameters in ensemble models which made SCC strength prediction more accurate. The mix of studying problems worldwide and fine-tuning them allows it to address *complex* optimization challenges in civil engineering.

### Mountain gazelle optimizer (MGO)

The Mountain Gazelle Optimizer^29^ is a nature-inspired optimization algorithm that mimics the survival strategies and movement patterns of mountain gazelles. These gazelles are known for their agility, speed, and the ability to navigate challenging terrains, which are effectively modeled to explore and exploit the search space. The algorithm uses adaptive mechanisms inspired by the gazelle’s behaviors, such as position updates, fitness evaluation, and speed-inspired exploration. These mechanisms are applied to the MGO algorithm to balance exploration and exploitation dynamically. The MGO integrates dynamic balancing between exploration and exploitation through adaptive parameters, ensuring convergence to optimal or near-optimal solutions. The algorithm focuses on refining high-potential solutions through localized movements within the search space. Maternity herds generate diverse candidate solutions, encouraging aggressive exploration and avoiding stagnation in suboptimal regions. Finally, migration for foraging allows long-range movements to explore underutilized regions, preventing the algorithm from being trapped in local optima. The mathematical representation of these behaviors includes adaptive weights, stochastic position updates, and fitness-based evaluation to guide the search process, mimicking the natural dynamics of mountain gazelles while ensuring computational efficiency. Literature can be referred for extensive mathematical formulation of the algorithm^30,31^.

### Fox optimizer

The Fox Optimizer^32^ is a biologically inspired algorithm that replicates the intelligent and adaptive behaviors of foxes, such as solitary hunting, group coordination, and territorial instincts. Solitary hunting (precision mechanism), group coordination (social mechanism), territorial instincts (dominance mechanism), and adaptive behavior (learning mechanism) are modeled in the Fox algorithm to perform localized exploitation, refine solutions in promising regions of the search space and maintain diversity. Dynamic adaptation employs adaptive mechanisms to balance exploration and exploitation based on the current state of the search, ensuring a steady progression toward global optima without premature convergence. The algorithm uses probabilistic rules to ensure intelligent candidates move intelligently towards global and local optima. The iterations are continued until a predefined stopping criterion is reached. More details regarding the mathematical formulation of the algorithm can be referred from the literature^32,33^.

### Brown bear optimizer algorithm (BBOA)

The Brown Bear Optimizer Algorithm (BBOA) is a nature-inspired metaheuristic based on the foraging, territorial, and hibernation behaviors of brown bears^34^. It simulates the bear’s adaptive strategies for survival across different seasons—switching between active exploration during food search and focused exploitation during rest or hibernation. BBOA introduces candidate solutions as bears that adjust their positions using two primary mechanisms: wide-range movement to explore new regions and localized refinement near high-potential areas. The algorithm adapts step size and movement intensity based on fitness feedback, mimicking seasonal adaptation and energy conservation. Its dynamic balance between diversification and intensification allows it to avoid local optima and converge toward global solutions. In this study, BBOA was employed for hyperparameter optimization of ensemble ML models, particularly Gradient Boosting, to enhance SCC compressive strength prediction. BBOA outperformed other optimizers in accuracy and robustness, demonstrating its suitability for solving complex engineering optimization problems.

## Data preparation and ML algorithms

### Data preprocessing

To develop an accurate and reliable machine learning model for predicting the compressive strength of self-compacting concrete (SCC), a comprehensive dataset comprising 691 samples was compiled from literature review of various research paper^35–50^. Each sample includes 9 input variables and 1 output variable (compressive strength). The dataset is consistent with several previous machine learning studies on SCC where mix composition-based datasets are used for strength prediction. Prior to model training, all input and output values were normalized to a 0–1 range using min–max scaling (Eq. 1). Normalization was necessary because the variables have different units and value ranges (for example, cement content in kg/m^3^ vs. age in days), and scaling them helps improve the training stability and accuracy of ML models. The min–max normalization is given by Eq. 1:

1 $${x}_{norm}=\frac{x-{x}_{min}}{{x}_{max}-{x}_{min}}$$

Here, *x*_min_ and *x*_max_ are the minimum and maximum values of the original parameter, *x*_x_ and *x*_norm_ are the actual and normalized values respectively. After normalization, the dataset was randomly split into a training set and a testing set to evaluate the model’s generalization ability. Following preprocessing, the dataset was split into training (80%) and testing (20%) subsets to evaluate the model’s generalization capability. A fixed random seed was used during data splitting to ensure reproducibility and prevent variability in results. To ensure robust model development and avoid overfitting, hyperparameter tuning using metaheuristic optimization was performed in conjunction with a tenfold cross-validation strategy on the training dataset. In this approach, the training data were partitioned into ten subsets, and the model was iteratively trained and validated to evaluate performance across folds. The average validation error was used as the objective function for optimization. This combined strategy enhances the reliability and generalization capability of the developed models. The training subset (552 samples) was used to develop machine learning models, while the testing subset (139 samples) provided an unbiased assessment of prediction accuracy. The test dataset was strictly kept unseen during model training and hyperparameter optimization to avoid data leakage. However, since the dataset was compiled from multiple literature sources, random splitting may introduce potential overlap of study-specific characteristics between training and testing datasets, which can lead to optimistic performance estimates. Future studies may consider grouped data splitting based on source studies or experimental campaigns for a more rigorous evaluation of model generalization.

The modeling workflow followed a strict sequence to avoid data leakage. First, the dataset was divided into training and testing subsets. Second, hyperparameter tuning using metaheuristic optimization was performed exclusively on the training dataset. Third, the selected model was trained using the optimized hyperparameters. Finally, the trained model was evaluated once on the unseen test dataset to assess its generalization performance. To ensure data quality and improve model performance, a preprocessing strategy was adopted. The dataset was examined for potential inconsistencies and extreme values using statistical indicators. In addition, the distribution of input variables was assessed using statistical indicators such as skewness and kurtosis. Advanced transformation techniques, including logarithmic and Box–Cox transformations, were examined to address potential skewness and stabilize variance. However, since the dataset exhibited acceptable distribution characteristics after normalization, these transformations were not applied in order to preserve the physical interpretability of the input parameters.

### Data curation and standardization

The dataset was compiled from multiple literature sources, where variations in reporting units and experimental standards were observed. To ensure consistency, all input variables were standardized to uniform units, including kg/m^3^ for material quantities, MPa for compressive strength, and days for curing age. Missing values were carefully examined, and only complete records were retained to ensure data integrity. The dataset was also screened for inconsistencies and redundancies, and duplicate or repeated mix designs reported across different studies were identified and removed to avoid bias in model training.

In addition, consistency checks were performed to ensure that all variables were within realistic physical ranges based on established concrete technology principles. These preprocessing steps ensured a clean, reliable, and standardized dataset suitable for machine learning modeling.

### Descriptive analysis and Pearson correlation

The dataset includes key influencing parameters (as shown in Table 1) such as cement content (ranging from 61 to 550 kg/m^3^, with a mean of 319.03 kg/m^3^), fly ash (0 to 378 kg/m^3^, mean: 144.10 kg/m^3^), slag (0 to 440 kg/m^3^, mean: 10.81 kg/m^3^), silica fume (0 to 250 kg/m^3^, mean: 7.42 kg/m^3^), water content (124.8 to 390.39 kg/m^3^, mean: 187.53 kg/m^3^), fine and coarse aggregate content, superplasticizer dosage, curing age (1 to 365 days, mean: 38.39 days), and the corresponding compressive strength values (4.44 to 105.8 MPa, mean: 40.07 MPa).The collected dataset underwent an extensive statistical analysis to understand the distribution of input parameters and their relationship with the target variable. Key statistical indicators, such as mean, standard deviation, skewness, and kurtosis, were computed to assess the variability and distribution of the data in Table 1. For example, the standard deviation of compressive strength was found to be 19.83 MPa, indicating significant variability in the dataset. Data preprocessing is a critical step to ensure the robustness and generalizability of machine learning models. Initially, missing values were handled using appropriate imputation techniques, and outliers were identified and addressed to avoid model bias.

**Table 1:** Descriptive analysis.

	_Cement_	_Fly Ash_	_Slag_	_SF_	_Water_	_FA_	_CA_	_SP)_	_Age_	_fck_
_Mean_	_319.03_	_144.10_	_10.81_	_7.42_	_187.53_	_838.52_	_802.89_	_5.06_	_38.39_	_40.07_
_Standard error_	_3.96_	_3.75_	_1.96_	_1.01_	_1.31_	_4.44_	_4.80_	_0.18_	_2.07_	_0.75_
_Median_	_315_	_160_	_0_	_0_	_182_	_851_	_837_	_3.255_	_28_	_39_
_Mode_	_250_	_0_	_0_	_0_	_178.6_	_786_	_837_	_6.5_	_28_	_46_
_Standard deviation_	_104.07_	_98.47_	_51.50_	_26.49_	_34.35_	_116.74_	_126.21_	_4.72_	_54.54_	_19.83_
_Sample variance_	_10,830_	_9696_	_2652_	_702_	_1180_	_13,627_	_15,930_	_22_	_2974_	_393_
_Kurtosis_	− _0.57_	− _0.76_	_32.57_	_31.84_	_3.75_	_2.28_	_0.84_	_2.87_	_20.26_	− _0.52_
_Skewness_	_0.25_	_0.04_	_5.47_	_5.02_	_1.31_	− _0.86_	_0.40_	_1.64_	_4.01_	_0.32_
_Range_	_489_	_378_	_440_	_250_	_265.59_	_740_	_650_	_20.955_	_364_	_101.36_
_Minimum_	_61_	_0_	_0_	_0_	_124.8_	_369_	_540_	_0_	_1_	_4.44_
_Maximum_	_550_	_378_	_440_	_250_	_390.3_	_1109_	_1190_	_20.9_	_365_	_105.8_
_Count_	_691_	_691_	_691_	_691_	_691_	_691_	_691_	_691_	_691_	_691_

**Fig. 2:**
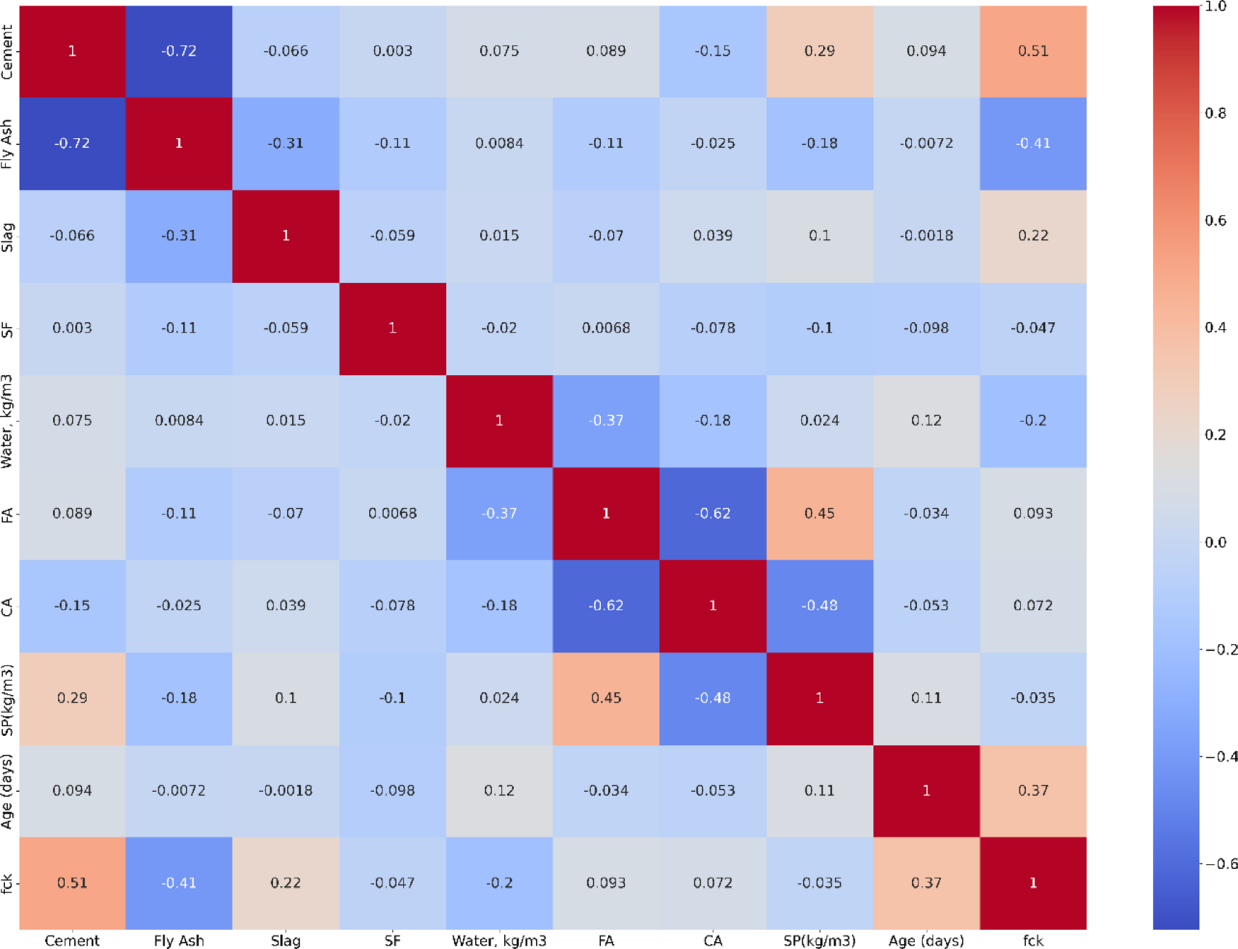
Pearson correlation.

The Pearson correlation matrix (Fig. 2) was evaluated to identify interdependencies between input variables and their impact on compressive strength. The Pearson correlation coefficient measures the linear relationship between variables and ranges from − 1 (strong negative correlation) to + 1 (strong positive correlation). In this study, cement content showed a strong positive correlation with compressive strength (r = 0.85), indicating that higher cement content generally leads to higher strength. Water content exhibited a moderate negative correlation (r = − 0.68), suggesting that increased water content reduces compressive strength due to increased porosity. Fly ash and slag had relatively weak correlations with compressive strength (r = 0.25 and r = 0.18, respectively), indicating a less direct influence compared to cement and water content. By implementing rigorous data processing and analysis techniques, we ensured that the machine learning models were trained on high-quality, meaningful data, ultimately leading to improved predictive performance and reliability.

## Results and discussion

### Hyperparameter tuning

The hyperparameter ranges considered in this study were selected based on established practices reported in the literature and preliminary experimental trials. Instead of conventional approaches such as grid search or random search, a metaheuristic optimization framework was adopted to efficiently explore the hyperparameter space. The optimization process was implemented using GWO, MGO, FOX and BBOA, with a population size of 50 and 100 iterations, as presented in Table 2. The objective function was defined to minimize the root mean square error (RMSE), ensuring optimal predictive performance. This approach offers a robust alternative to traditional tuning methods by enhancing global search capability and reducing the likelihood of convergence to local optima, while also lowering computational effort. The optimized hyperparameters obtained through each metaheuristic algorithm are summarized in Table 2. The results indicate that the BBOA-based model achieved the lowest RMSE value of 3.9428, outperforming GWO, MGO, and FOX as shown in Table 3. These findings demonstrate that metaheuristic-driven hyperparameter tuning significantly improves model accuracy and provides an efficient and practical solution for predictive modelling in engineering applications.

**Table 2:** Hyperparameter of metaheuristic algorithm.

Hyperparameter	Range	Best solution (i.e., Hyperparameters)			
		GWO	MGO	FOX	BBOA
learning_rate	[0.01, 0.3]	0.1221	0.1624	0.1165	0.2109
n_estimators	[50, 1000]	432	315	398	325
max_depth	[3,10]	4	4	4	4
subsample	[0.5, 1]	0.8268	0.8841	0.6057	0.9025

**Table 3:** Statistical results for training.

Training							
	Random Forest	AdaBoost	Gradient Boosting	GWO	MGO	FOX	BBOA
R^2^	0.9803	0.8613	0.9478	0.9923	0.9832	0.9887	0.9955
WMAPE	0.0474	0.1541	0.0825	0.0326	0.0465	0.0392	0.0246
NS	0.9803	0.8613	0.9478	0.9924	0.9832	0.9887	0.9956
RMSE	2.74	7.27	4.46	1.71	2.53	2.07	1.30
VAF	98.03	86.53	94.78	99.23	98.32	98.87	99.55
PI	− 0.779	− 5.506	− 2.562	0.276	− 0.564	− 0.095	0.686
WI	0.9948	0.9548	0.9860	0.9930	0.9845	0.9896	0.9989

### Performance evaluation of predicting compressive strength

The evaluation of predictive models is an essential step in determining their effectiveness, reliability, and suitability for a given application. Performance metrics provide quantitative measures to assess how well a model captures the relationships in the data and makes accurate predictions. It’s important to evaluate the performance of a ML model through multiple statistical indices. Each metric provides a unique perspective, addressing different aspects of model performance (e.g., fit, variance, error distribution, agreement). The study utilizes 7 metrices for the performance evaluation of all the ML models simulated in the study: Coefficient of Determination (R^2^), Weighted Mean Absolute Percentage Error (WMAPE), Nash–Sutcliffe Efficiency (NS), Root Mean Squared Error (RMSE), Variance Accounted For (VAF), Prediction Index (PI) and Willmott’s Index of Agreement (WI)^51–55^. R^2^ measures the proportion of variance in the observed data that is explained by the model’s predictions. Ranging from 0 to 1,1 indicates a strong correlation between predicted and observed values, while a 0 indicates the model does not better than the mean of the observations. R^2^ is widely used for its simplicity and interpretability, but does not account for overfitting or outliers. WMAPE is a metric used to express the absolute error of a model as a percentage of the total observed values. It is suitable for datasets with large variations in magnitudes and is easy to interpret. The Nash–Sutcliffe Efficiency coefficient evaluates the predictive performance of a model relative to the mean of the observed values. It ranges from -∞ to 1, with an ideal value of 1 indicating no error in predictions. RMSE measures the average magnitude of prediction errors, giving more weight to larger errors. VAF measures the percentage of variance explained by the model, with an ideal value of 100%. PI quantifies the improvement of a model over a baseline predictor, with an ideal value of 100% demonstrating a significant reduction in prediction error. WI assesses the agreement between observed and predicted values, with an ideal value of 1, indicating perfect match between the two. The mathematical formulations of the matrices are given from Eq. 2 to 8.

2 $${R}^{2}=\frac{\sum_{i=1}^{n}{\left({d}_{i}-{d}_{avg}\right)}^{2}-\sum_{i=1}^{n}{\left({d}_{i}-{y}_{i}\right)}^{2}}{\sum_{i=1}^{n}{\left({d}_{i}-{d}_{avg}\right)}^{2}}$$

3 $$WMAPE=\frac{\sum_{i=1}^{n}\left|\frac{{d}_{i}-{y}_{i}}{{d}_{i}}\right|\times {d}_{i}}{\sum_{i=1}^{n}{d}_{i}}$$

4 $$NS=1-\frac{{\sum}_{i=1}^{n}{({d}_{i}-{y}_{i})}^{2}}{{\sum}_{i=1}^{n}{({d}_{i}-{d}_{mean})}^{2}}$$

5 $$RMSE=\sqrt{\frac{1}{n}\sum_{i=1}^{n}{\left({d}_{i}-{y}_{i}\right)}^{2}}$$

6 $$VAF=\left(1-\frac{var\left({d}_{i}-{y}_{i}\right)}{var({d}_{i}{y}_{i})}\right)\times 100$$

7 $$PI=adj.{R}^{2}+\left(0.01\times VAF\right)-RMSE$$

8 $$WI=1-\left[\frac{\sum_{i=1}^{n}{\left({d}_{i}-{y}_{i}\right)}^{2}}{\sum_{i=1}^{n}{\left\{\left|{y}_{i}-{d}_{avg}\right|+\left|{d}_{i}-{d}_{avg}\right|\right\}}^{2}}\right]$$

The values of the metrices are given in Table 3. The highest value of R^2^ is for BBOA (0.9955), indicating it explains 99.55% of the variance in the training data, making it the most accurate model during training. AdaBoost (0.8613) has the lowest R^2^, suggesting it performs the worst among these models in terms of fit. BBOA has the lowest RMSE, indicating the smallest overall error, while AdaBoost has the highest RMSE, indicating the largest errors. BBOA achieved the highest PI (0.686), showing the most improvement, while AdaBoost (− 5.506) had the weakest variance explanation. BBOA (0.9989) achieves the highest WI, reflecting nearly perfect agreement. AdaBoost (0.9548) has the lowest WI, showing weaker agreement. In testing as well in Table 4, BBOA achieves the highest R^2^ (0.9644), indicating excellent generalization, while AdaBoost has the lowest R^2^ (0.8150). BBOA reports has the lowest WMAPE (0.0743), highest NS (0.9644) and lowest RMSE (3.943). Noteworthy, AdaBoost has the worst PI (− 7.326) which indicates that it is performing worse than the baseline. Models like GWO, MGO, and FOX show strong results but are slightly less effective compared to BBOA. The consistent dominance of BBOA across training and testing datasets suggests strong performance. A large performance gap between training and testing datasets is a strong indicator of overfitting. there is no large gap between the training and testing performance for the best-performing models, overfitting is not evident. For the best-performing model (BBOA), R^2^ is 0.9955 for training and 0.9644 for testing, with a small difference (~ 0.031), indicating good generalization to unseen data. The models seem to generalize well to unseen data.

**Table 4:** Statistical results for training.

Testing							
	Random Forest	Ada Boost	Gradient Boosting	GWO	MGO	FOX	BBOA
R^2^	0.8931	0.8150	0.9091	0.9446	0.9482	0.9492	0.9644
WMAPE	0.1255	0.1958	0.1255	0.0943	0.0930	0.0908	0.0743
NS	0.8931	0.8150	0.9091	0.9446	0.9482	0.9492	0.9644
RMSE	6.84	8.99	6.30	4.92	4.76	4.71	3.94
VAF	89.38	82.15	90.91	94.46	94.82	94.93	96.45
PI	− 5.0529	− 7.326	− 4.4898	− 3.037	− 2.867	− 2.817	− 2.016
WI	0.9698	0.9372	0.9746	0.9859	0.9867	0.9865	0.9909

**Fig. 3:**
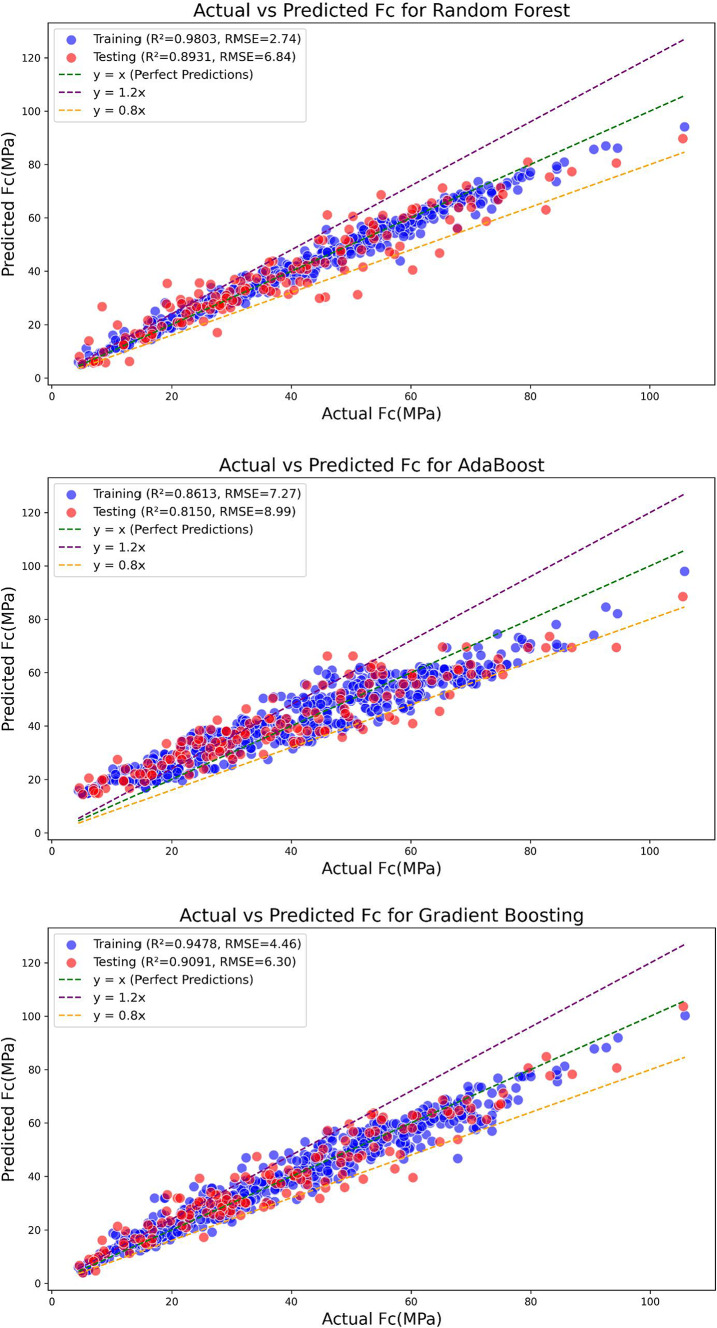

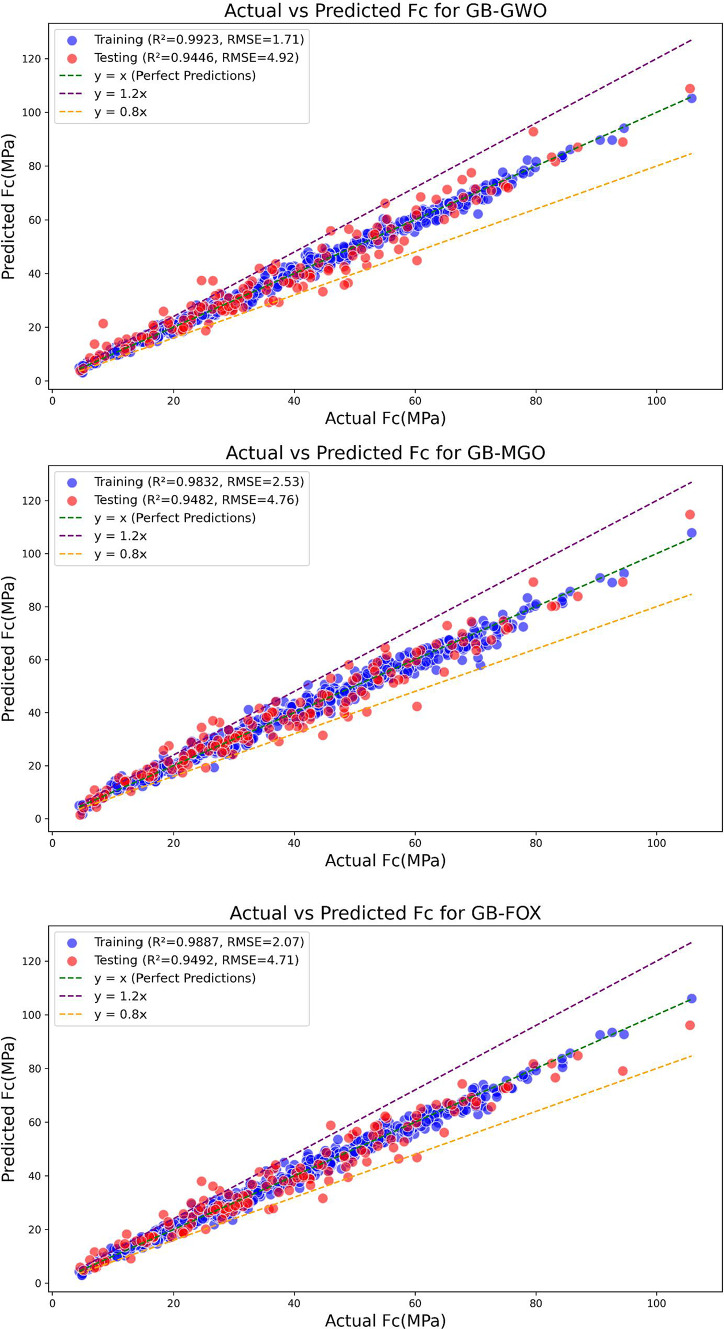

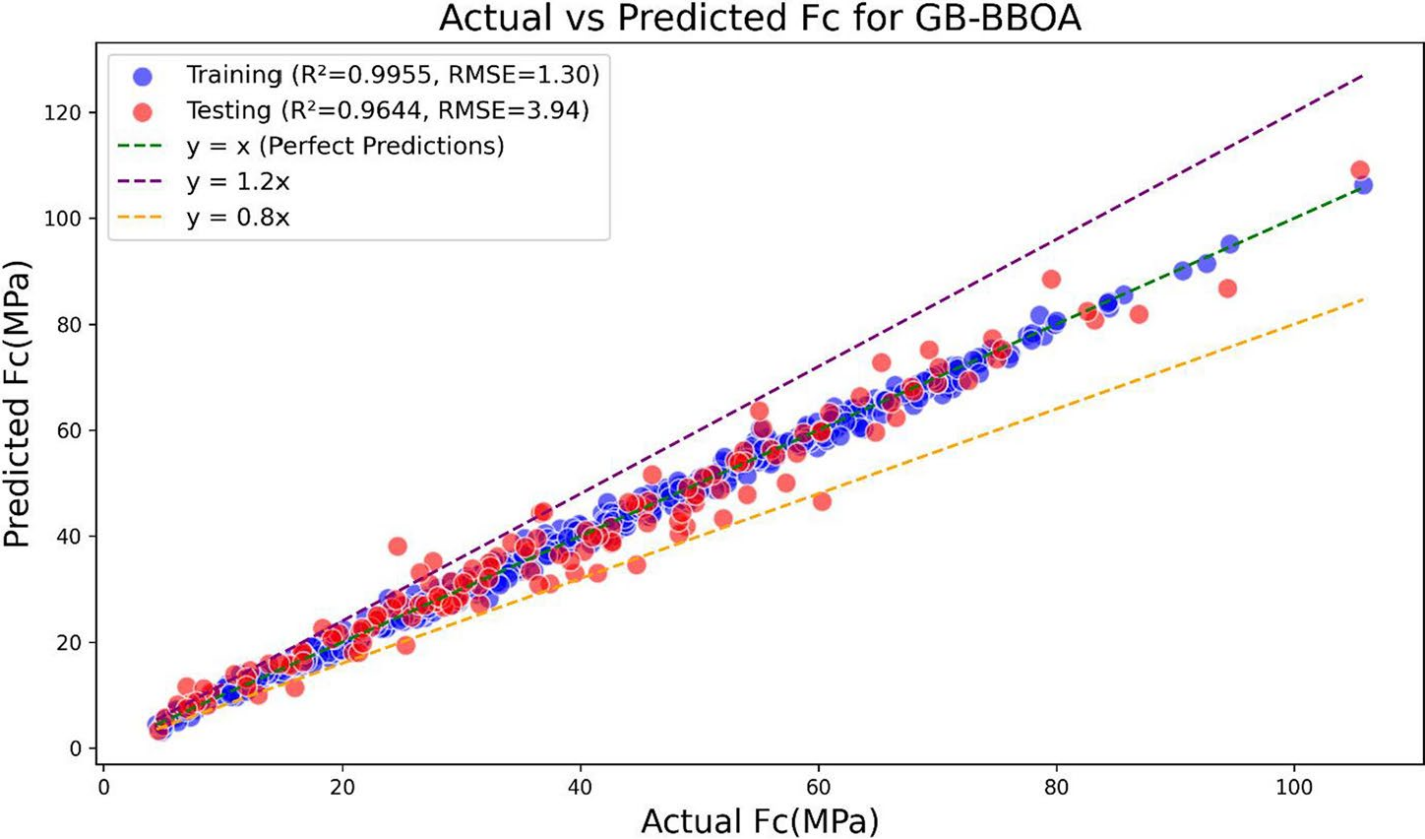
Actual versus predicted graphs for developed models.

Figure 3 presents actual vs predicted scatter plots of all the models developed in the study. An actual vs. predicted scatter plot is a crucial diagnostic tool used in predictive modeling to evaluate the performance of regression models^56–58^. It compares the actual values of the target variable against the predicted values generated by the model, providing insights into the model’s predictive capabilities and potential areas for improvement. The purpose of this plot is to assess the model’s performance by comparing the closer points lie to the diagonal line (y = x) or perfect predictions line, which indicates perfect data fitting. Insights from the plot include a well-fitting model, underfitting, overfitting, heteroscedasticity, nonlinear relationships, and outliers. The plots show the performance of each model in predicting F_c_, with the X-axis representing actual values and the Y-axis representing predicted values. Data points are blue and red, with solid black lines representing perfect predictions and dashed lines representing overprediction and underprediction. The BBOA model demonstrates the best predictive ability, followed by FOX Optimizer. Random Forest exhibits signs of overfitting (better training but lower testing performance). AdaBoost shows consistent performance but at a lower overall accuracy compared to others.

### SHAP analysis

**Fig. 4:**
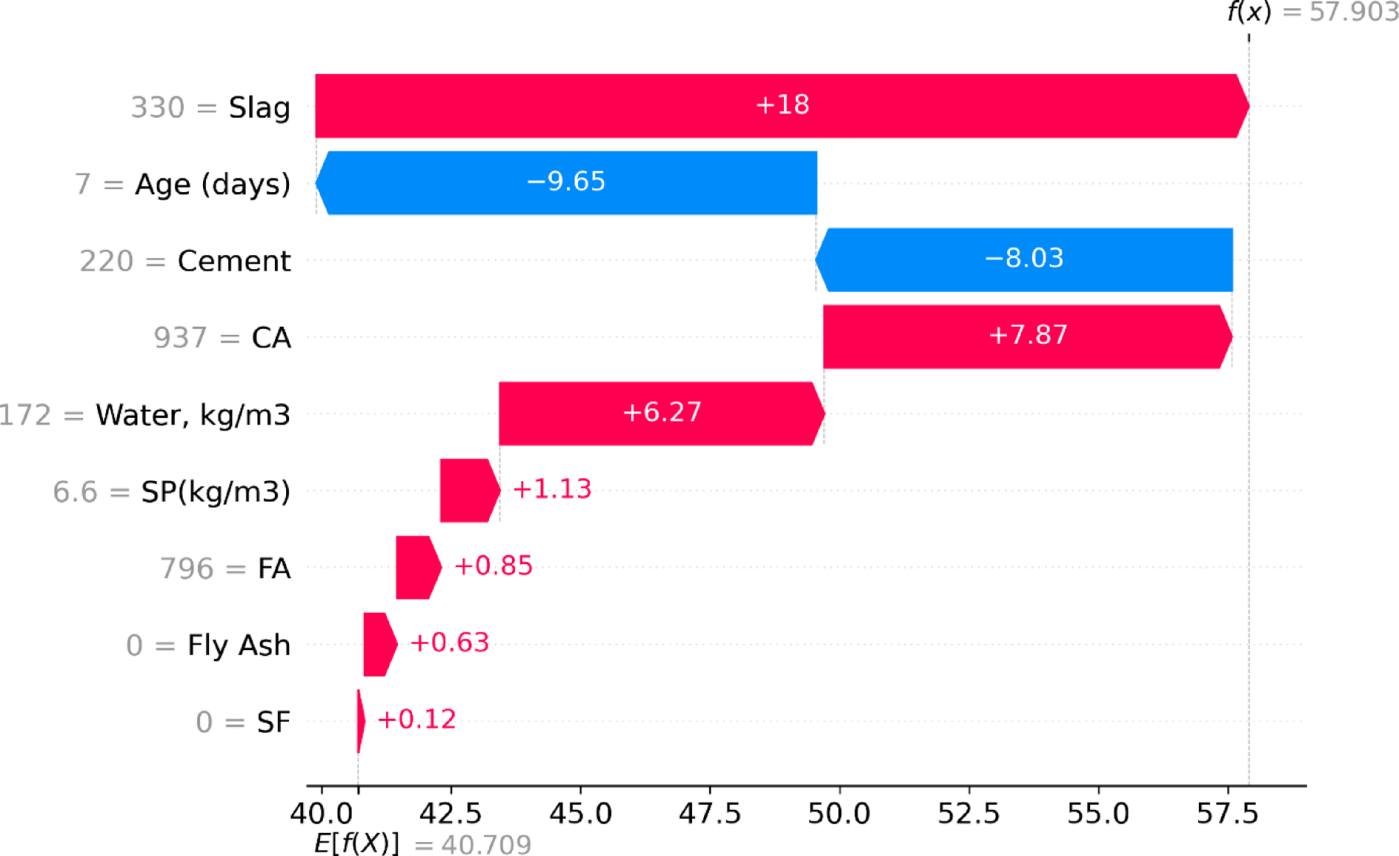
Waterfall chart.

In this study, SHAP (Shapley Additive Explanations) was employed to interpret the predictions of the GB–BBOA model. SHAP analysis provides practical insights for mix design optimization by identifying key influencing parameters such as slag content, cement content, and curing age. Practitioners can utilize these insights to prioritize critical variables, reduce trial-and-error experimentation, and achieve efficient and cost-effective SCC mix design^59^. SHAP analysis was performed using the TreeExplainer method, which is specifically designed for tree-based models. The SHAP values were computed using the trained model and evaluated on the test dataset to ensure unbiased interpretation of feature importance. The background dataset was derived from the training data distribution, enhancing the reliability and transparency of the interpretation. The SHAP framework enables detailed understanding of feature contributions by quantifying the impact of each input variable on model predictions. The generated visualizations, including summary plots and dependency plots, reveal both linear and nonlinear relationships between input features and compressive strength. The waterfall chart (Fig. 4) illustrates the cumulative contribution of each feature from the baseline value to the final prediction. The baseline value, representing the average model output, is 40.709. Among the input parameters, slag content shows the highest positive contribution (+ 18), while curing age (7 days) and cement content (220 kg/m^3^) exhibit negative contributions of − 9.65 and − 8.03, respectively. Coarse aggregate and water content contribute positively, with values of + 7.87 and + 6.27, respectively. Other parameters, including superplasticizer, fine aggregate, fly ash, and silica fume, show comparatively smaller contributions. The final predicted compressive strength is 57.903.

Overall, SHAP analysis revealed that slag content, cement content, and curing age are the most significant features influencing SCC compressive strength prediction.

**Fig. 5:**
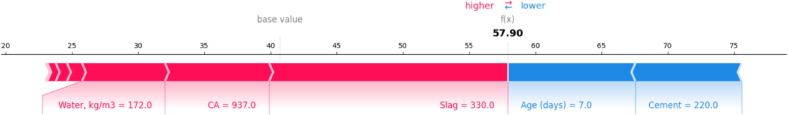
Force Map.

This information is further substantiated by the force map in Fig. 5 (which emphasizes the features’ ideal values) to maximize the predicted outcome. The baseline value—indicative of the model’s expected prediction across the dataset—is approximately 40. The final predicted value for this specific instance is 57.90; the deviation from the baseline arises because of the influence of various features. Among these, Water (172 kg/m^3^), Coarse Aggregate (CA = 937) and Slag (330) demonstrate the most significant positive impacts, thus pushing the prediction higher. However, it is crucial to analyze these contributions carefully (to understand their roles in the overall prediction). On the other hand, features, Age (7 days) and Cement (220) substantially decrease the prediction. This force plot effectively illustrates how the combination of these feature contributions results in the final prediction. The visualization enhances interpretability by providing insights into the relative importance and directionality of each feature’s impact, which is critical for understanding model behavior in data-driven research. It should be noted that the SHAP values presented in the waterfall plot correspond to a local explanation for a specific sample and do not represent global monotonic relationships between input variables and compressive strength. For instance, although curing age generally has a positive influence on strength development, its negative contribution in this example reflects the local interaction of features for the selected sample rather than a general trend.

### Taylor diagram presentation for ML models

**Fig. 6:**
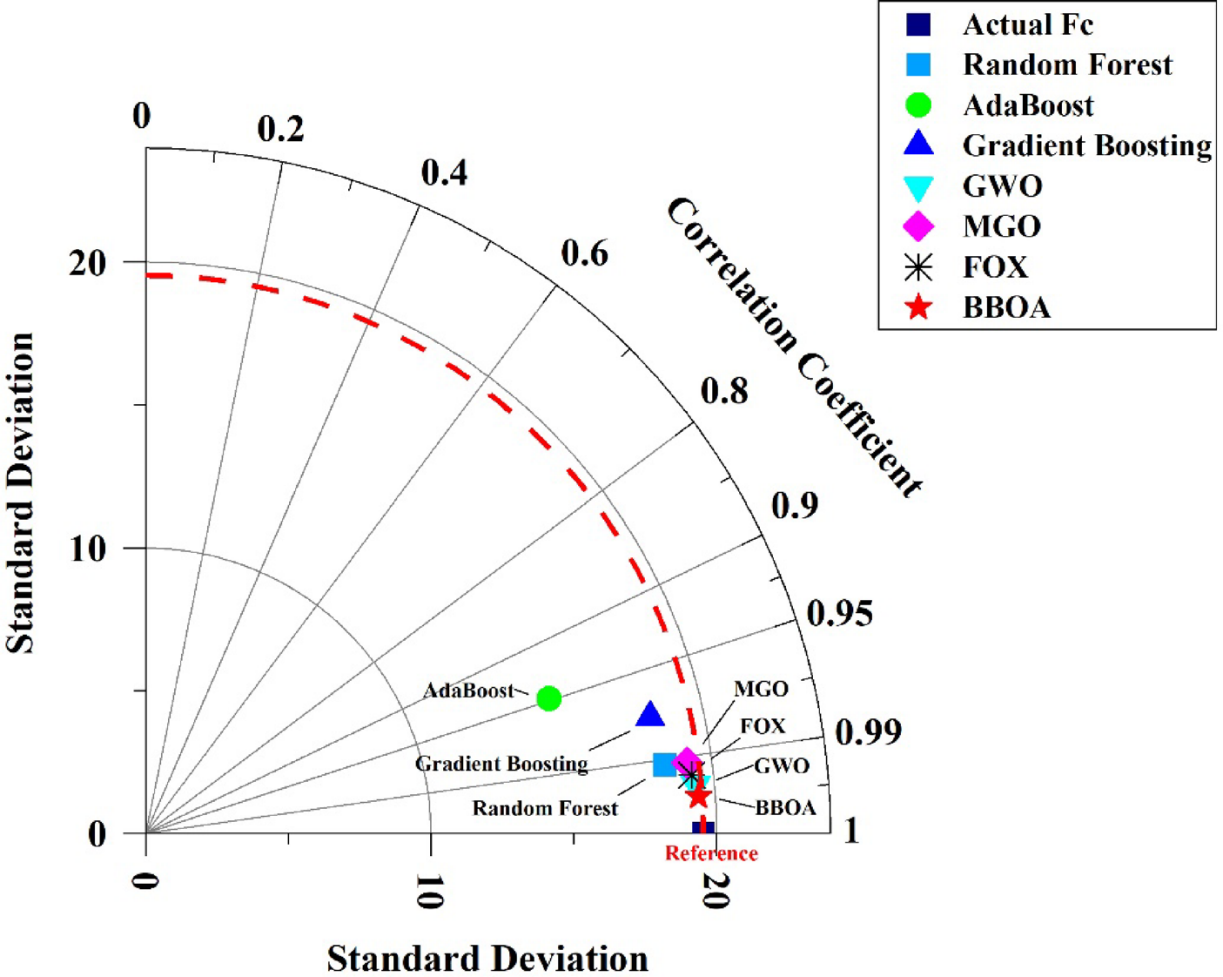
Taylor diagram for training dataset.

**Fig. 7:**
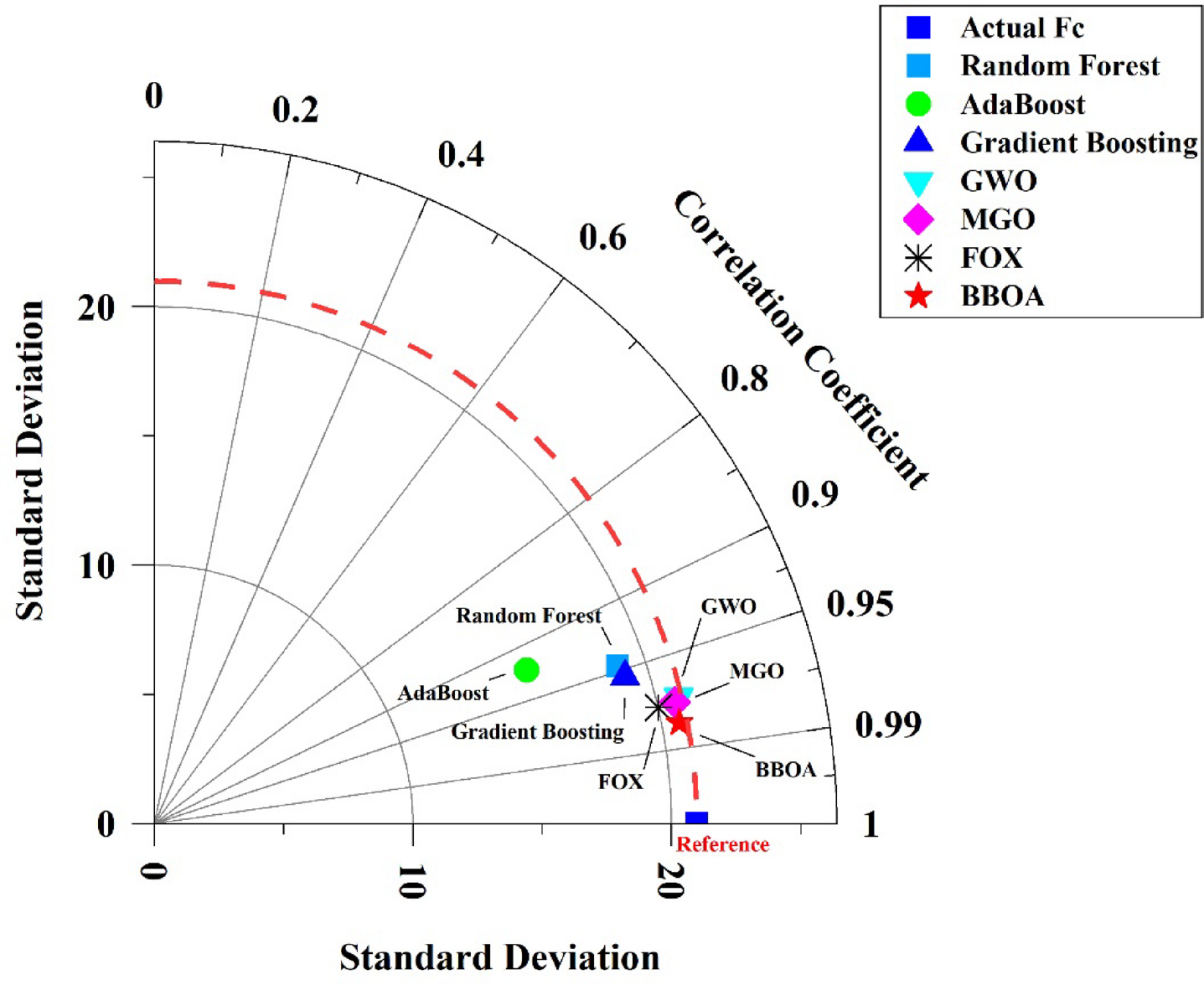
Taylor diagram for testing dataset.

The Taylor Diagram is a specialized graphical tool designed to assess the performance of various models or methods in relation to a reference dataset. It simultaneously represents key statistical metrics, including the correlation coefficient, standard deviation, and root mean square error (RMSE), within a unified framework. The primary components of the diagram include: (1) the radial distance, which denotes the standard deviation of the modeled data; (2) the angular coordinate, which corresponds to the correlation coefficient between the model and the reference dataset; and (3) the distance from the reference point, which indicates the proximity to ideal performance. The plot in Fig. 6 and 7 reveals that MGO, FOX, GWO, and BBOA are closely clustered near the reference point, demonstrating strong alignment with the reference dataset and effective modeling of variability. The Random Forest model is positioned slightly farther from the reference point compared to these models but still exhibits relatively good performance, characterized by a high correlation coefficient and a standard deviation close to the reference. In contrast, Gradient Boosting and AdaBoost are located at greater distances from the reference point, indicating lower correlation coefficients and higher deviations, which suggest inferior performance relative to the other models.

### Statistical testing

Statistical testing is essential for assessing the reliability and robustness of machine learning models used in predicting the compressive strength of self-compacting concrete (SCC). The uncertainty analysis was conducted at a 95% confidence level using the statistical distribution of prediction errors^59–61^. The standard deviation and standard error were used to compute the confidence bounds, providing a reliable estimate of model variability. In this study, we conducted an uncertainty analysis (UA) to evaluate the prediction errors of the developed models. The error (εᵢ) between the actual (oᵢ) and predicted (ôᵢ) outputs was computed using Eq. 9:

9 $$\varepsilon_{{\mathrm{i}}} = \left| { o_{{\mathrm{i}}} - \hat{o}_{{\mathrm{i}}} } \right|$$

The mean absolute error (MAE) was first calculated as:

10 $$MAE=\frac{1}{n}\sum_{i=1}^{n}{\varepsilon}_{i}$$

From these errors, key statistical measures, including the standard deviation (SD) and standard error (SE), were derived as follows in Eq. 11 and 12:

11 $$SD = \sqrt{\sum_{i=1}^{n}{\left({\varepsilon}_{i}-\overline{\varepsilon }\right)}^{2}}$$

12 $$SE = \frac{SD }{\sqrt{n}}$$

The margin of error (ME) at the 95% confidence level was determined as:

13 $$ME=1.96\times SE$$

A confidence interval (CI) was determined using the margin of error (ME) at a 95% confidence level, which was subsequently used to compute the upper bound (ub), lower bound (lb), and width of confidence bound (WCB) in Eq. 12:

14 $$ub = MAE + ME, lb = MAE - ME, WCB = ub - lb$$

The WCB represents the range within which approximately 95% of the prediction errors are expected to fall, indicating the reliability of each model. The uncertainty bounds were estimated based on prediction errors obtained during model evaluation, ensuring a reproducible and consistent assessment of model performance.

The results of the UA for all the machine learning models are summarized in Table 5. The BBOA model exhibited the lowest WCB (6.937) and mean absolute error (MAE) (2.888), demonstrating its superior reliability compared to other models. Furthermore, models such as MGO (WCB: 6.808, MAE: 3.617), GWO (WCB: 6.654, MAE: 3.668), and FOX (WCB: 6.876, MAE: 3.529) showed competitive performance with relatively low WCB values, whereas AdaBoost had the highest WCB (5.177) and MAE (7.611), indicating higher uncertainty in its predictions. For a comprehensive comparison, models were ranked based on their WCB values. In cases where models exhibited the same WCB, additional criteria such as MAE and SD were used for ranking. The results revealed that the BBOA model consistently outperformed other models, achieving the first rank across all evaluation criteria. A comparative analysis with existing predictive models highlights the robustness of the proposed hybrid machine learning approach in accurately estimating SCC compressive strength.

**Table 5:** Uncertainty analysis.

	N	MAE	STD D	ME	UB	LB	WCB	Rank
Training								
BBOA	552	0.995	19.41	1.620	2.614	− 0.625	3.239	1
GWO	552	1.317	19.34	1.614	2.930	− 0.297	3.227	2
FOX	552	1.583	19.23	1.605	3.188	− 0.022	3.210	3
MGO	552	1.876	19.13	1.596	3.472	0.280	3.192	4
RF	552	1.913	18.35	1.531	3.444	0.382	3.062	5
GB	552	3.329	18.14	1.514	4.843	1.816	3.027	6
ADA	552	6.221	14.90	1.243	7.464	4.978	2.487	7
Testing								
BBOA	139	2.888	20.86	3.468	6.357	− 0.580	6.937	1
FOX	139	3.529	20.68	3.438	6.967	0.091	6.876	2
MGO	139	3.617	20.47	3.404	7.021	0.212	6.808	3
GWO	139	3.668	20.01	3.327	6.995	0.341	6.654	4
RF	139	4.878	19.07	3.171	8.049	1.707	6.342	5
GB	139	4.877	18.91	3.145	8.022	1.733	6.290	6
ADA	139	7.611	15.56	2.588	10.199	5.023	5.177	7

**Fig. 8:**
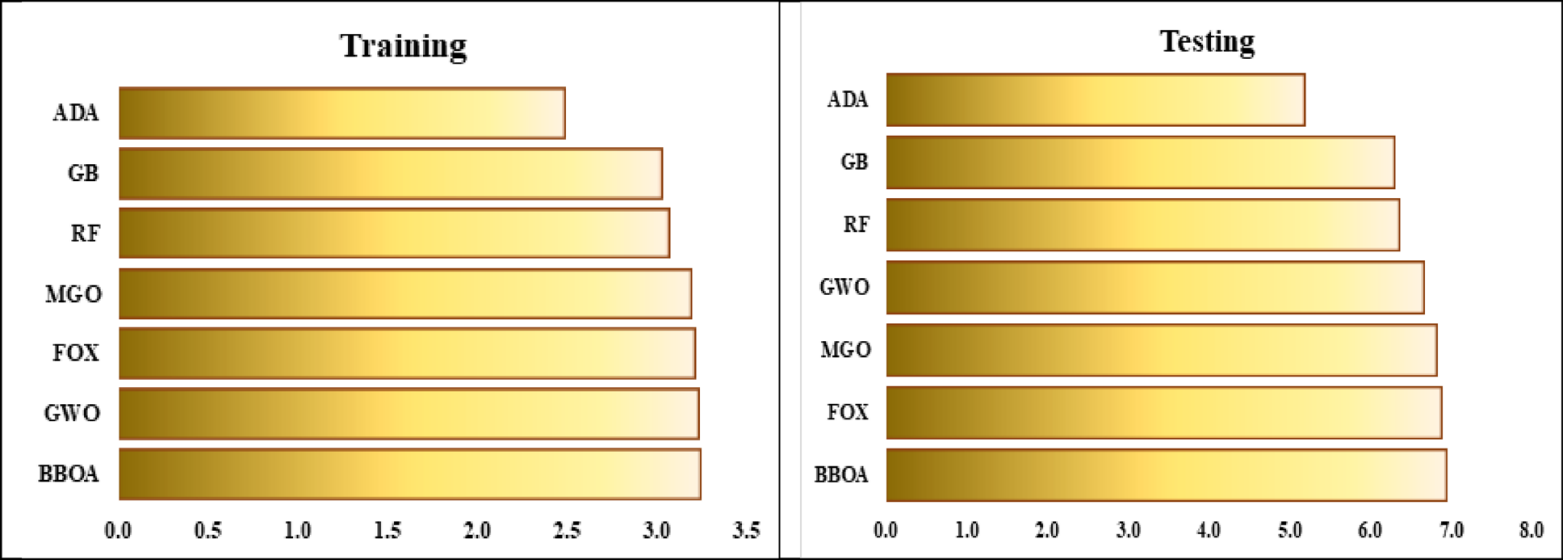
Comparative analysis of Uncertainty analysis in training and testing.

The findings of the statistical testing reinforce the reliability of the developed predictive models, particularly the hybrid BBOA model, in real-world engineering applications. The results confirm that advanced machine learning techniques, coupled with optimization strategies, enhance the predictive accuracy and generalizability of SCC strength estimation models. The comparative results are illustrated in Fig. 8, which clearly shows the superior performance of the BBOA model over other tested algorithms.

### Error line plot

**Fig. 9:**
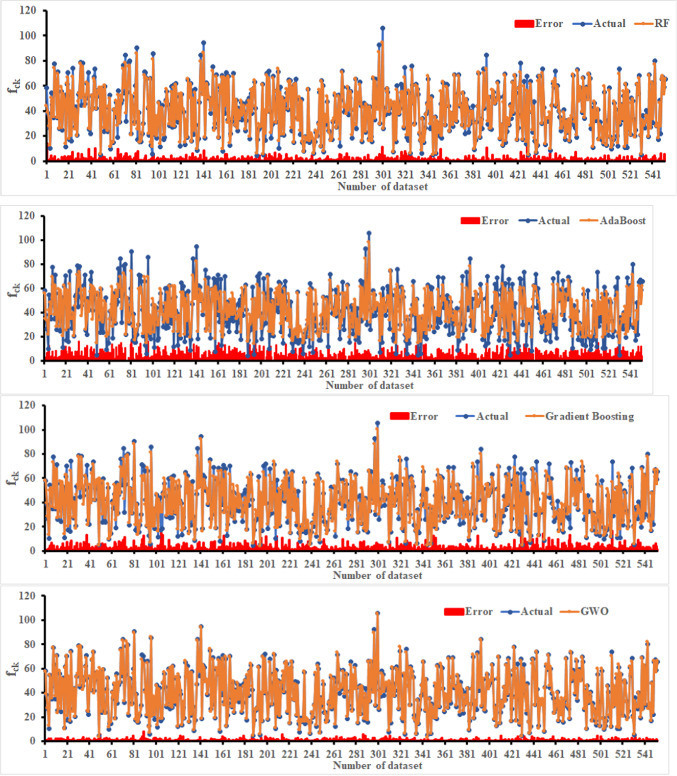

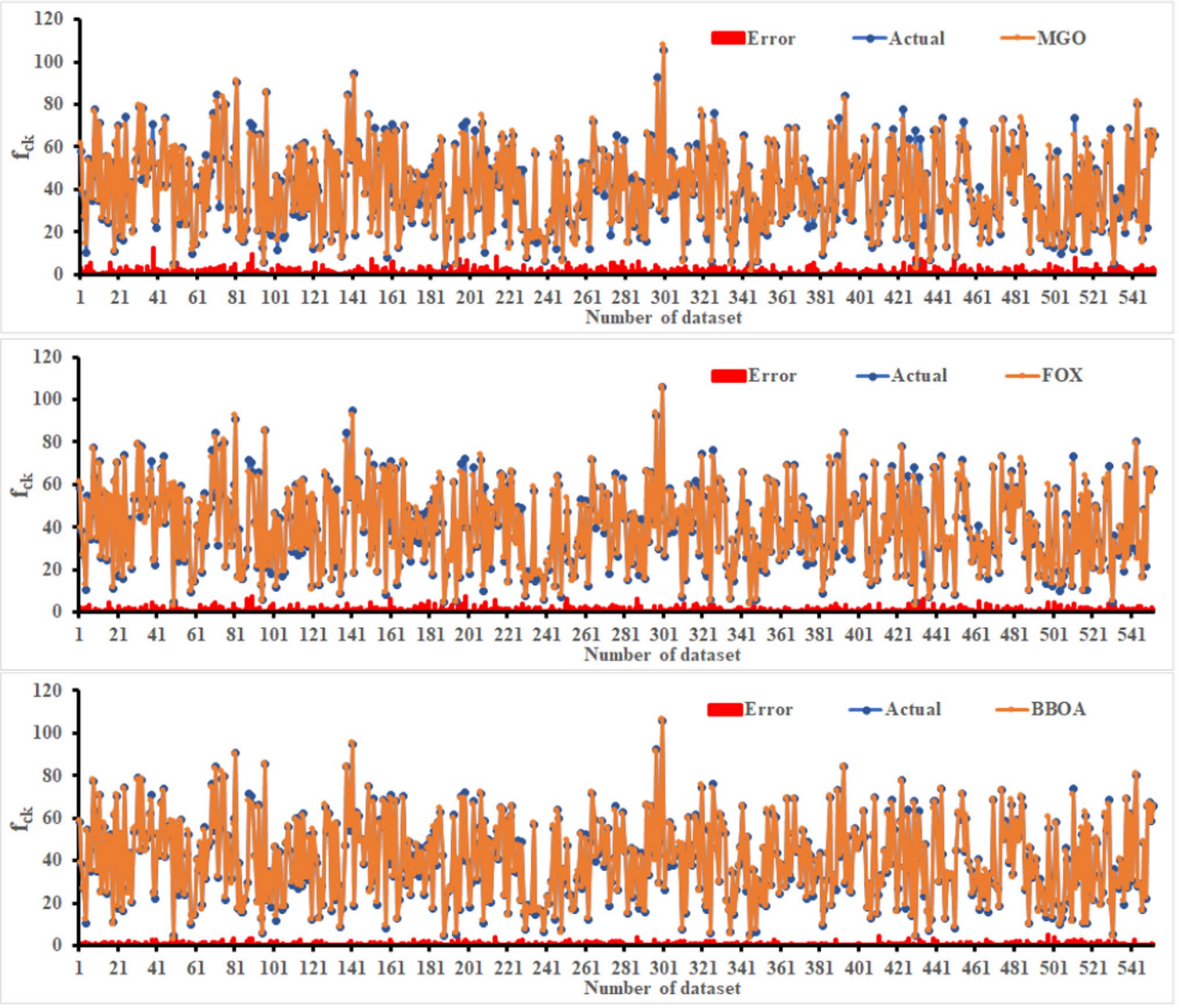
Error plot of actual and predicted result of developed of model.

**Fig. 10:**
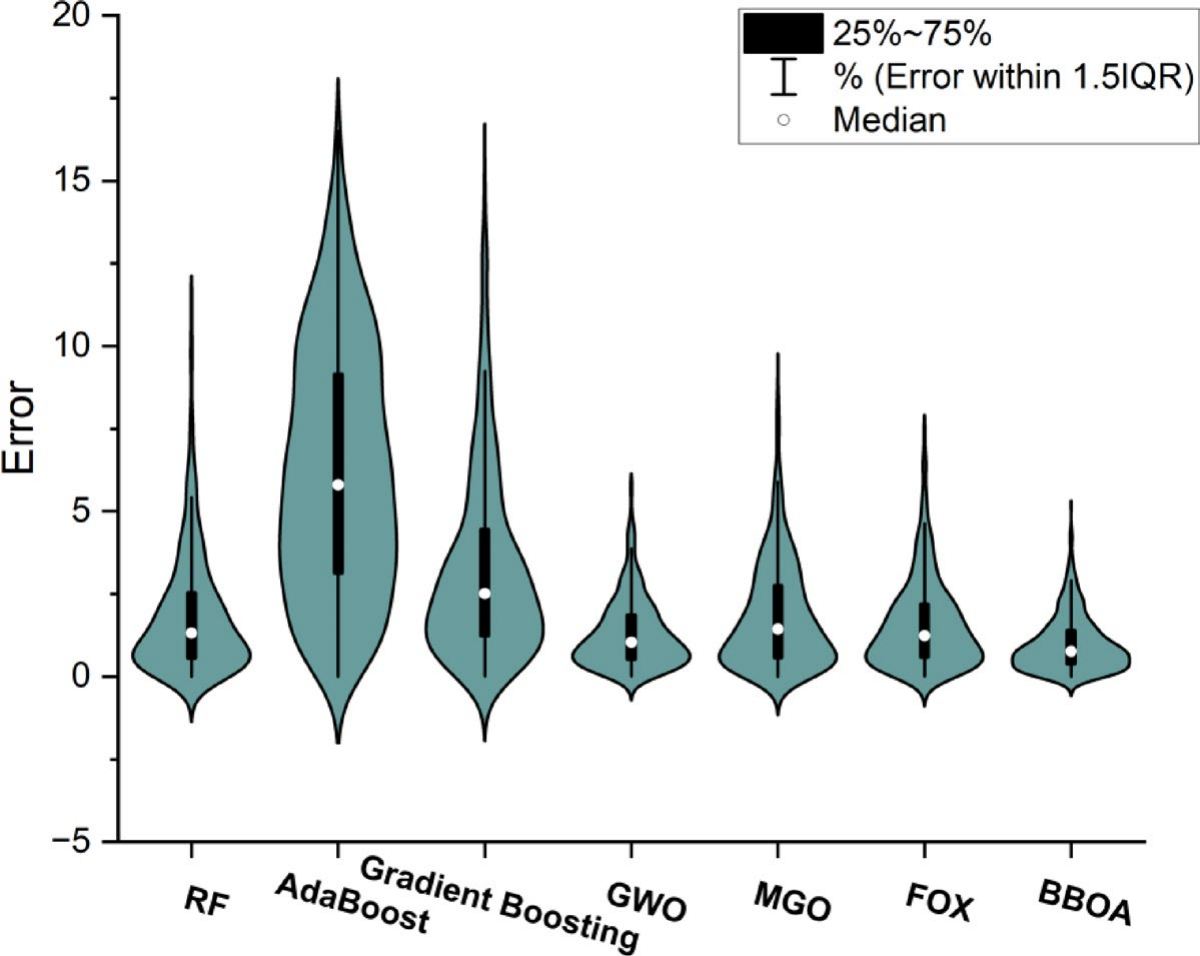
Violin plot.

Error line plots are a powerful visualization tool that combine line graphs and error representations, commonly used in data analysis, experimental research, and predictive modeling. They help visualize model predictions, highlight variability, evaluate uncertainty, and enable direct comparisons of performance. It includes a central line representing actual values, error bars representing uncertainty, and axes and labels. Benefits include intuitive representation, clear trend analysis, and emphasis on uncertainty^62–66^. Figure 9 presents error line plots for the ML models developed in the study. BBOA performs well for most datasets, with the predicted line aligning closely with the actual values. AdaBoost and gradient boosting follows the actual values closely, but there may be slightly more variation in errors compared to other models. The quantity of errors in AdaBoost is very high, which makes it an unreliable model. GWO and FOX models also shows satisfactory performances. Figure 9 presents violin plot distribution for the comparative analysis of errors of the models. It provides a detailed view of how errors are distributed, including the median, interquartile range (IQR), and overall error spread. Key elements of the violin plot include the violin shape, median, interquartile range, whiskers, and outliers. The plot shows that RF has a narrow and consistent error distribution with a relatively small IQR, indicating it produces reliable and low error predictions. AdaBoost shows a wider violin and larger IQR compared to RF, suggesting higher variability and larger error values. Gradient Boosting displays the widest violin shape, indicating a highly variable error distribution and generally higher errors compared to other models. GWO, MGO, FOX, and BBOA have very narrow and concentrated violins, suggesting extremely consistent predictions with very low error values. FOX and BBOA appear to have the smallest errors overall. Median comparisons show that FOX and BBOA are the most promising models due to their low and consistent error distributions, making them suitable for applications where minimizing prediction errors is critical. BBOA exhibit the smallest and most consistent error distributions, making it the best performers in terms of prediction accuracy. The results can be concluded from the violin plot depicted in Fig. 10.

### Graphical user interface for practical engineering

**Fig. 11:**
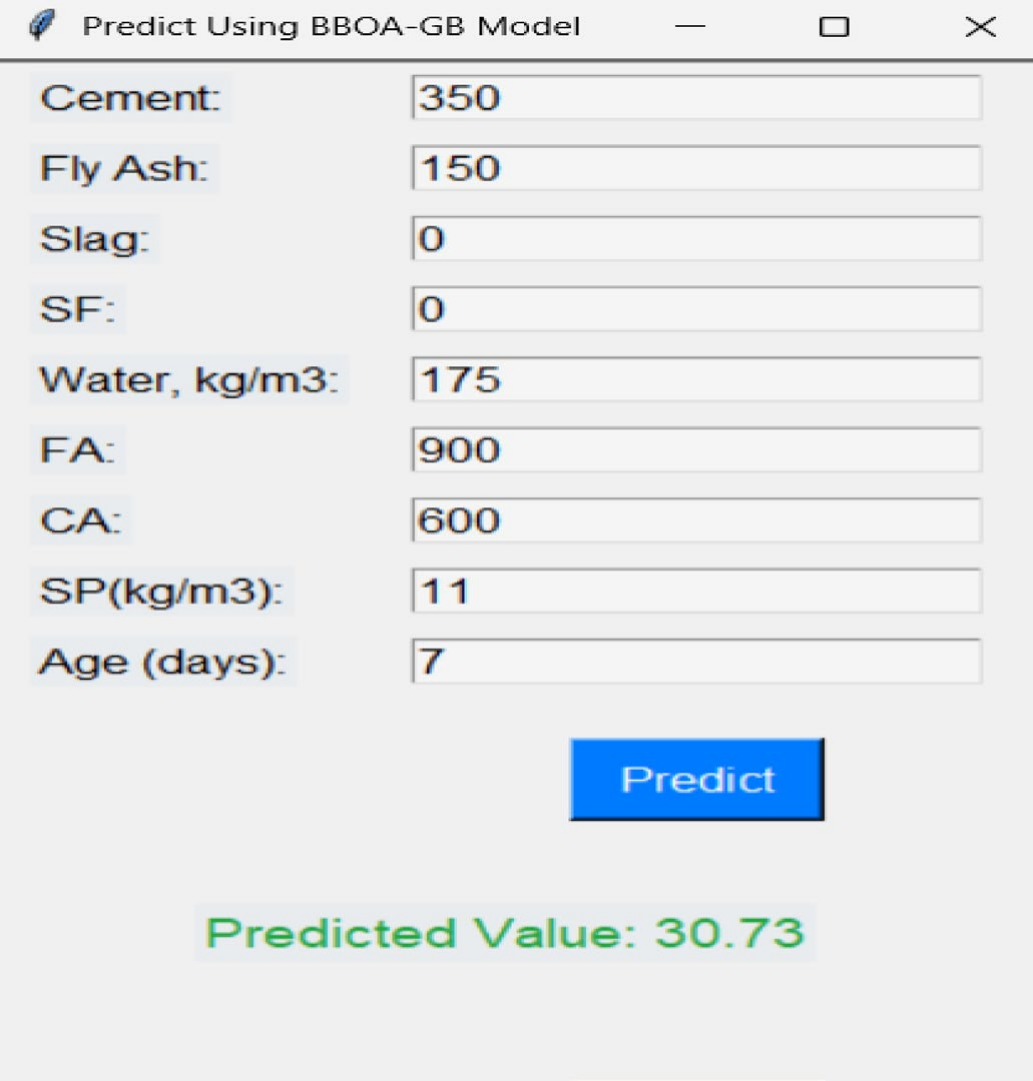
GUI of BBOA-GB.

The growing dependence on machine learning (ML) for predictive analysis in concrete design necessitates a balance (this) between data-driven models and empirical methods to ensure practical relevance for design engineers. Although ML techniques offer high predictive accuracy, their practical implementation presents several challenges: data quality, feature selection, model interpretability and generalization across diverse datasets. Inconsistent (or) limited experimental data can hinder model performance; selecting the most influential input parameters significantly impacts prediction reliability. Furthermore, ML models often function as black boxes (however), which limits their interpretability—crucial for engineering applications. Overfitting can also affect generalization, making it difficult to apply trained models to real-world scenarios because of this. To tackle these challenges, this study presents the BBOA-GB model, a highly efficient ML approach for estimating compressive strength of Self-Compacting Concrete (SCC). To improve accessibility and usability, we have crafted a user-friendly graphical user interface (GUI) (as shown in Fig. 11), accessible at https://github.com/AkhilendraSharma/SCC-BBOA-GB, which allows users to enter key mix design parameters—such as cement, water, superplasticizer, fly ash, silica fume, coarse and fine aggregates and concrete age—on a per cubic meter basis. The GUI processes these inputs swiftly, providing predictions of compressive strength. However, because of the complexity involved, users must ensure accuracy in their inputs. Although this interface is designed to be intuitive, some users may still encounter difficulties. Its intuitive design ensures that users (with varying technical expertise) can engage with the model without needing programming skills, making it suitable for both engineers and researchers. However, this accessibility doesn’t undermine the model’s sophisticated capabilities; rather, it enhances its usability. Although some may argue that simplicity compromises depth, the reality is that it invites a broader audience to the field of machine learning (ML). Because of this, the potential for innovative applications expands significantly. Additionally, the interface is optimized for efficiency: it delivers accurate results with minimal computational load, facilitating quick assessments of mix design feasibility. Transparency of the system allows users to visualize both the input parameters and corresponding predictions (fostering a deeper understanding) of the relationship between mix design and concrete performance. Through the integration of interactive visualization tools and real-time feedback mechanisms, the GUI significantly enhances the adoption (and trustworthiness) of ML-driven compressive strength prediction; ultimately contributing to improved concrete design practices. However, this system is not devoid of limitations, because there are always challenges in adapting to user needs. Although it is designed to be user-friendly, some users might struggle initially.

However, with time (and practice), individuals can master the interface. This is because consistent effort leads to improvement, although some might find it challenging at first. Ultimately, mastery is achievable. The developed machine learning framework offers significant practical advantages for real-world SCC mix design. By accurately predicting compressive strength, the proposed model can reduce the need for extensive trial-and-error experimentation, leading to savings in time, labor, and material costs. Furthermore, the identification of key influencing parameters enables more efficient use of cementitious materials, contributing to reduced carbon footprint and improved sustainability. These insights support data-driven decision-making and facilitate the development of cost-effective and environmentally optimized SCC mixtures for practical engineering applications.

## Conclusions

This study presented an advanced ML-based framework for predicting the compressive strength of SCC using hybrid ensemble models optimized through metaheuristic algorithms. Among the developed models, BBOA-GB exhibited superior predictive accuracy and generalization capability. The integration of metaheuristic optimization with SHAP analysis ensured high performance and interpretability, establishing the framework as a reliable and explainable prediction tool.

Comparative benchmarking of four metaheuristic optimizers within the hybrid ensemble framework provided valuable insights into their relative efficiency in optimizing ensemble algorithms. Although no new algorithm was proposed, the systematic evaluation of hybrid configurations enhanced understanding of metaheuristic–ensemble interactions and their role in SCC mix optimization. The developed GUI further strengthened the study’s practical relevance by enabling user-friendly, real-time predictions, thereby bridging the gap between data-driven modeling and engineering application. Key findings demonstrated that BBOA-GB achieved the highest predictive accuracy with R^2^ values of 0.9955 and 0.9645 in training and testing, respectively, and the lowest RMSE of 1.305 MPa and 3.943 MPa. Feature analysis identified slag content, cement content and curing age as the most significant parameters influencing SCC strength. Taylor diagram and uncertainty analyses confirmed the superior reliability of BBOA-GB, with the lowest WCB of 6.937 during testing. Overall, the proposed hybrid ML framework offers a robust, interpretable, and sustainable approach for SCC strength prediction and contributes to advancing intelligent, data-driven design strategies in modern concrete technology.

However, the present study is limited by the use of a literature-based dataset compiled from previously published experimental studies, which may restrict the generalizability of the developed models to SCC mixtures outside the considered parameter range. In addition, the proposed framework has been validated on a single compiled database and may benefit from further verification using larger, more diverse, and real-time datasets. Future research should focus on expanding the dataset to include a broader range of SCC mix proportions and curing conditions, integrating real-time sensor data for dynamic prediction, and developing deep learning-based models for enhanced accuracy. The present study focuses on selected ensemble models (RF, AdaBoost, and Gradient Boosting) integrated with metaheuristic optimization. Although these models provide a strong basis for comparison, other advanced machine learning algorithms such as XGBoost, LightGBM, CatBoost, support vector regression, and neural networks were not included. Future work will extend the proposed framework to incorporate these models under a unified evaluation protocol for comprehensive benchmarking. Enhancing the GUI with cloud-based capabilities will enable real-time collaboration and wider adoption in the construction industry. In conclusion, this research underscores the transformative potential of machine learning in concrete technology, offering a data-driven, efficient and scalable approach to SCC strength prediction. The study’s findings suggest that ML-driven models, particularly hybrid approaches optimized with metaheuristics, can drastically improve prediction accuracy, reduce material testing costs and streamline construction processes. The insights gained from this study contribute to advancing the field of smart construction materials, enabling better-informed engineering decisions that align with sustainability and performance objectives.

## Data Availability

The datasets used and/or analysed during the current study available from the corresponding author on reasonable request.
